# Topical dura mater application of CFA induces enhanced expression of c-fos and glutamate in rat trigeminal nucleus caudalis: attenuated by KYNA derivate (SZR72)

**DOI:** 10.1186/s10194-017-0746-x

**Published:** 2017-03-23

**Authors:** M. Lukács, K. Warfvinge, J. Tajti, F. Fülöp, J. Toldi, L. Vécsei, L. Edvinsson

**Affiliations:** 1 Department of Clinical Sciences, Division of Experimental Vascular Research, Lund University, Lund, Sweden; 2 Department of Clinical Experimental Research, Copenhagen University, Glostrup Hospital, Copenhagen, Denmark; 3 Department of Neurology, University of Szeged, 6725 Semmelweis street nr. 6, Szeged, Hungary; 4 Institute of Pharmaceutical Chemistry and MTA-SZTE Research Group for Stereochemistry, University of Szeged, Szeged, Hungary; 5 Department of Physiology, Anatomy and Neuroscience, University of Szeged, Szeged, Hungary; 6 MTA SZTE Neuroscience Research Group, Szeged, Hungary

**Keywords:** TNC, CFA, c-fos, Glutamate, KYNA analogue

## Abstract

**Background:**

Migraine is a debilitating neurological disorder where trigeminovascular activation plays a key role. We have previously reported that local application of Complete Freund’s Adjuvant (CFA) onto the dura mater caused activation in rat trigeminal ganglion (TG) which was abolished by a systemic administration of kynurenic acid (KYNA) derivate (SZR72). Here, we hypothesize that this activation may extend to the trigeminal complex in the brainstem and is attenuated by treatment with SZR72.

**Methods:**

Activation in the trigeminal nucleus caudalis (TNC) and the trigeminal tract (Sp5) was achieved by application of CFA onto the dural parietal surface. SZR72 was given intraperitoneally (i.p.), one dose prior CFA deposition and repeatedly daily for 7 days. Immunohistochemical studies were performed for mapping glutamate, c-fos, PACAP, substance P, IL-6, IL-1β and TNFα in the TNC/Sp5 and other regions of the brainstem and at the C_1_-C_2_ regions of the spinal cord.

**Results:**

We found that CFA increased c-fos and glutamate immunoreactivity in TNC and C_1_-C_2_ neurons. This effect was mitigated by SZR72. PACAP positive fibers were detected in the fasciculus cuneatus and gracilis. Substance P, TNFα, IL-6 and IL-1β immunopositivity were detected in fibers of Sp5 and neither of these molecules showed any change in immunoreactivity following CFA administration.

**Conclusion:**

This is the first study demonstrating that dural application of CFA increases the expression of c-fos and glutamate in TNC neurons. Treatment with the KYNA analogue prevented this expression.

## Background

Migraine is among the leading causes of disability, having a huge impact on public health [1, 2]. Studies show that each year 2.5% of episodic migraine disease converts into chronic migraine [3] which appears as a distinct entity in the classification of the International Headache Society. Although numerous studies have been performed aiming to understand the pathophysiology of migraine and the chronification process, however this is still enigmatic. The trigeminal system plays a pivotal role in the genesis of migraine headache [4, 5]. The pseudo-unipolar nerve cells of the trigeminal ganglion (TG), provide sensory innervation of cranial structures and meningeal vessels while central projections terminate in trigeminal nucleus caudalis (TNC) and C_1_-C_2_ region of the spinal cord [6]. This trigeminovascular complex transmits pain signals from meningeal and cerebral vessels to the brainstem and second-order neurons terminate in the thalamus and cortical regions, where further transmission and modulation of pain sensation occur [6–8]. Following continous and repeated stimulation peripheral and central sensitisation of the primary-neurons might occur, leading to reduced activation treshold, represented clinically by allodynia [4, 9, 10].

Previous studies have shown that application of inflammatory substances on the dura mater causes central sensitisation of the neurons in TNC and at C_1_-C_2_ levels of the spinal cord [6, 10, 11]. Recent studies have demonstrated lower levels of kynurenic acid (KYNA) in serum of patients suffering from chronic migraine compared to controls [12, 13]. KYNA could be a new therapeutic line in migraine chronification, but KYNA can poorly cross the blood–brain barrier (BBB), while newer KYNA analogues have better BBB penetration characteristics [14, 15]. We have recently developed an animal model for long-term trigeminovascular activation following application of Complete Freund’s Adjuvant (CFA) onto the surface of the dura mater [16]. We found activation of satellite glial cells and neurons of the trigeminal ganglion (IL-1, pERK1/2) that were abolished by the KYNA-analogue, SZR72 [17] possibly acting on peripheral and central gluatamate receptors (30). The present study was designed to examine whether dural application of CFA can cause activation of the central part of the trigeminalvascular system,: the TNC and C_1_-C_2_ regions of the spinal cord. We asked the question whether the CFA-induced activation might be mitigated by use of SZR72 intraperitoneally.

## Methods

### Synthesis of novel KYNA derivative

The KYNA amide reported here was designed in the Pharmaceutical Chemistry and Research Group for Stereochemistry, University of Szeged Hungary. The synthesis procedure has previously been presented [15, 17]. The KYNA analogue (SZR72, N-(2-N,N-dimethylaminoethyl)-4-oxo-1H-quinoline-2-carboxamide hydrochloride) has the following structural properties: the presence of a water-soluble side-chain, the inclusion of a new cationic centre, and side-chain substitution in order to enhance brain penetration [17].

### Animals

Adult male Sprague–Dawley rats (220–300 g) (*n =* 30) were used. The animals were raised and maintained under standard laboratory conditions with free access to food and tap water. The study followed the guidelines of the European Communities Council (86/609/ECC) and was approved by the Ethics Committee of The Faculty of Medicine, University of Szeged, Hungary (I-74-12/2012).

### Treatments

The animals were divided into 7 groups: (i) CFA + saline application to the dura, (ii) saline application to the dura, (iii) pre-treatment KYNA (KYNA analog, 300 mg/kg body weight dissolved in 1 ml saline, 1 h before CFA administration), (iv) pre-treatment saline (saline, 1, ml 1 h before CFA), (v) repeated treatment (KYNA analog, 300 mg/kg body weight dissolved in 1 ml saline every 12 h, for 7 days), (vi) repeated saline (saline 1 ml every 12 h, for 7 days) and (vii) fresh (intact, control rats) (Table 1).

**Table 1:** Groups of animals

Group	Dura application	Pre-treatment	Repeated treatment	No. of animals
CFA	CFA	-	-	6
Saline	saline	-	-	3
CFA + KYNA pre-treatment	CFA	KYNA	-	6
CFA + saline pre-treatment	CFA	saline	-	3
CFA + KYNA repeated	CFA	KYNA	KYNA	6
CFA + saline repeated	CFA	saline	saline	3
Fresh, control rats	-	-	-	3

### Operation

The operation has been described in details earlier [16, 17]. Briefly, animals were deeply anesthetized and a handheld drill was used to remove a 3x3 mm large portion of the parietal bone, cooled by saline irrigation to avoid local healing. The hole was made postero-laterally to the bregma (5 mm), on the left side, care being taken not to penetrate the dura mater. Ten μl of CFA (Sigma-Aldrich, St. Louis, MO, USA) or saline was applied on the dural surface, and washed with saline after 20 min.

Both treated and control animals were transcardially fixation-perfused with 4% paraformaldehyde in buffer after 7 days. As fresh control, intact rats were used.

### Tissue analysis

After the perfusion-fixation the TNC brainstem region and C_1_-C_2_ region of the spinal cord were removed (−1, +5 mm from the obex). Specimens were frozen on dry ice, stored at −80 °C and prepeared for immunohistochemistry. To encompass TNC, sections were collected from 6 different levels from the central canal was visualized to the C_1_ segment of the spinal cord. (100–120 sections in total per animal).

### Immunohistochemistry and microscopic analysis

Immunohistochemical staining was performed to demonstrate the localization of glutamate, c-fos, TNF-α, IL-1β, IL-6, substance P and PACAP. Details of the primary and secondary antibodies are given in Table 2 and 3.

**Table 2:** Details of primary antibodies used for immunohistochemistry

Name	Product code	Host	Dilution	Company
Anti c-fos	PC38	Rabbit	1:100	Merck Millipore, Darmstadt, Germany
Anti PACAP-38	B57–1	Rabbit	1:100	Europroxima, Arnhem, Netherlands
Anti Glutamate	G9282	Mouse	1:100	Sigma-Aldrich, St-Luis, MO, USA
Anti Glutamate	AB5018	Rabbit	1:100	Merck Millipore, Darmstadt, Germany
Anti Substance P	B 45–1	Rabbit	1:200	Europroxima, Arnhem, Netherlands
Anti IL-1β	ab 9787	Rabbit	1:100	Abcam, Cambridge, UK
Anti IL-6	ab6672	Rabbit	1:200	Abcam, Cambridge, UK
Anti-TNF α	ab66579	Rabbit	1:400	Abcam, Cambridge, UK

**Table 3:** Details of secondary antibodies used for immunohistochemistry

Conjugate and host	Against	Diluation	Company
FITC (goat)	anti-rabbit	1:100	Cayman Chemical, Ann Arbor, MI, USA
Alexa 488 (goat)	anti-mouse	1:100	Invitrogen, CA, USA
Alexa 594 (donkey)	anti-rabbit	1:100	Jackson Immuno Research, West Baltimor, PA, USA

The immunohistochemical method and the microscopic analysis have been described earlier [16]. The areas of the brainstem were identified using rat brain atlas (Paxinos and Watson, second edition, 1986). Each procedure was repeated a minimum of three times to validate the results and minimize any experimental errors using the same antibody stock. Negative controls were performed for each set by omitting the primary antibody. One examinator was blinded. Any resulting immunofluorescence would suggest unspecific binding of the secondary antibodies.

## Results

### Immunohistochemistry

#### Glutamate

**Fig. 1:**
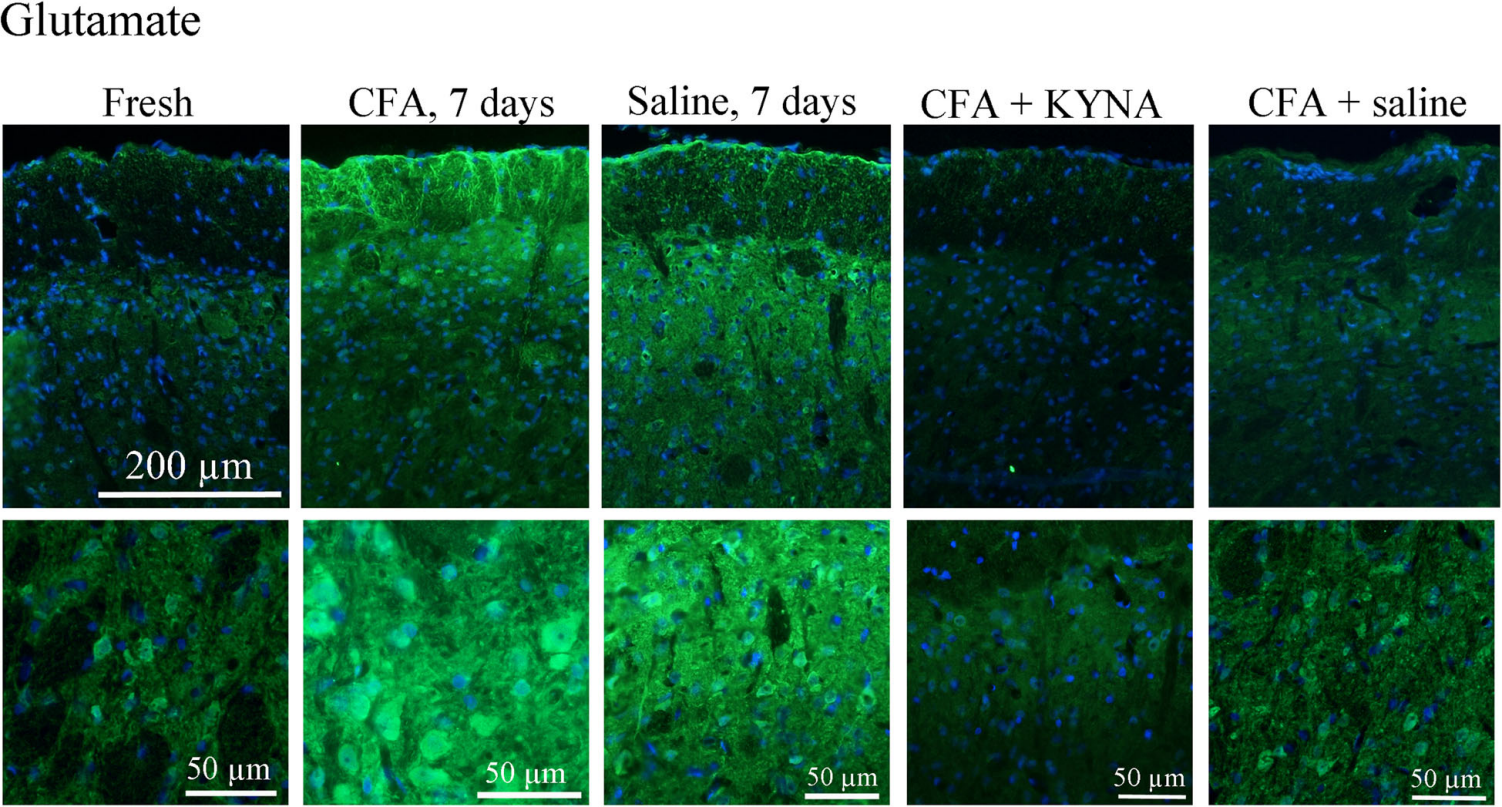
Glutamate immunoreactivity in the TNC. In case of fresh, intact animals a few homogenously stained neurons were detected in the TNC. Following CFA application on the dura a clear increase in staining intensity and amount of glutamate positive cells can be seen. After saline application on the dura a moderate increase was seen compared to fresh, intact animals. I.p. treatment with KYNA derivate abolished the amount of glutamate positive cells following CFA induced activation, whereas i.p saline treatment had no effect on glutamate reactivity in the TNC. “+” represent a light, “++” moderate, “+++” strong increase in immunoreactivity

In intact (fresh) animals, glutamate immunoreactivity (GI) was detected in fibers of the trigeminal tract on every level of the TNC (Fig. 1a and b). A few homogeneously stained glial cells could also be found. The staining also displayed some homogenously labelled neurons, especially in TNC in the caudal part of the brainstem.

Application of CFA on the dura (group CFA 7days), similar staining pattern was observed, with an obvious increase in the intensity and amount of glutamate positive neurons in the TNC (Fig. 1). In the gelatinous layer a clearly increased intensity could be seen. With higher magnification, cells with intensely stained cytoplasm were identified in this region (Fig. 1). The aspect is specific to the medial part of the spinal trigeminal nucleus: triangular or multipolar shaped, medium-sized cells with an irregular arrangement. No difference in the fiber staining was noted. In the application of saline group, the same GI pattern was observed (Fig. 1).

In group pretreated with SZR72, the increased expression was abolished. The intensity and number of glutamate immunoreactive cells remained at the level observed in healthy, intact animals. No clear difference could be visualized between pre-treatment and repeated-treatment of KYNA, and no difference was noted in the fibers and glial cells. In the application of saline group, the same GI pattern was observed as in fresh control (Fig. 1).

On the level of the C_1_-C_2_ region of the spinal cord GI was found in the anterior and dorsal horns (lamina I, lamina II) and the areas surrounding the central canal. In these areas no difference was found between the different groups (no cells, only fibers). A summary of these results is presented in Table 4.

**Table 4:** Summary of glutamate immunostaining in TNC for different treatment groups

Group	Neuronal staining	Fiber staining	Glial cell staining
Fresh	-\+	++	-\+
CFA 7d	+++	++	-\+
Saline 7d	+	++	-\+
CFA+ SZR72 one dose	-\+	++	-\+
CFA+ SZR72 repeated	-\+	++	-\+
CFA+ saline one dose	+++	++	-\+
CFA + saline repeated	+++	++	-\+

#### C-fos

**Fig. 2:**
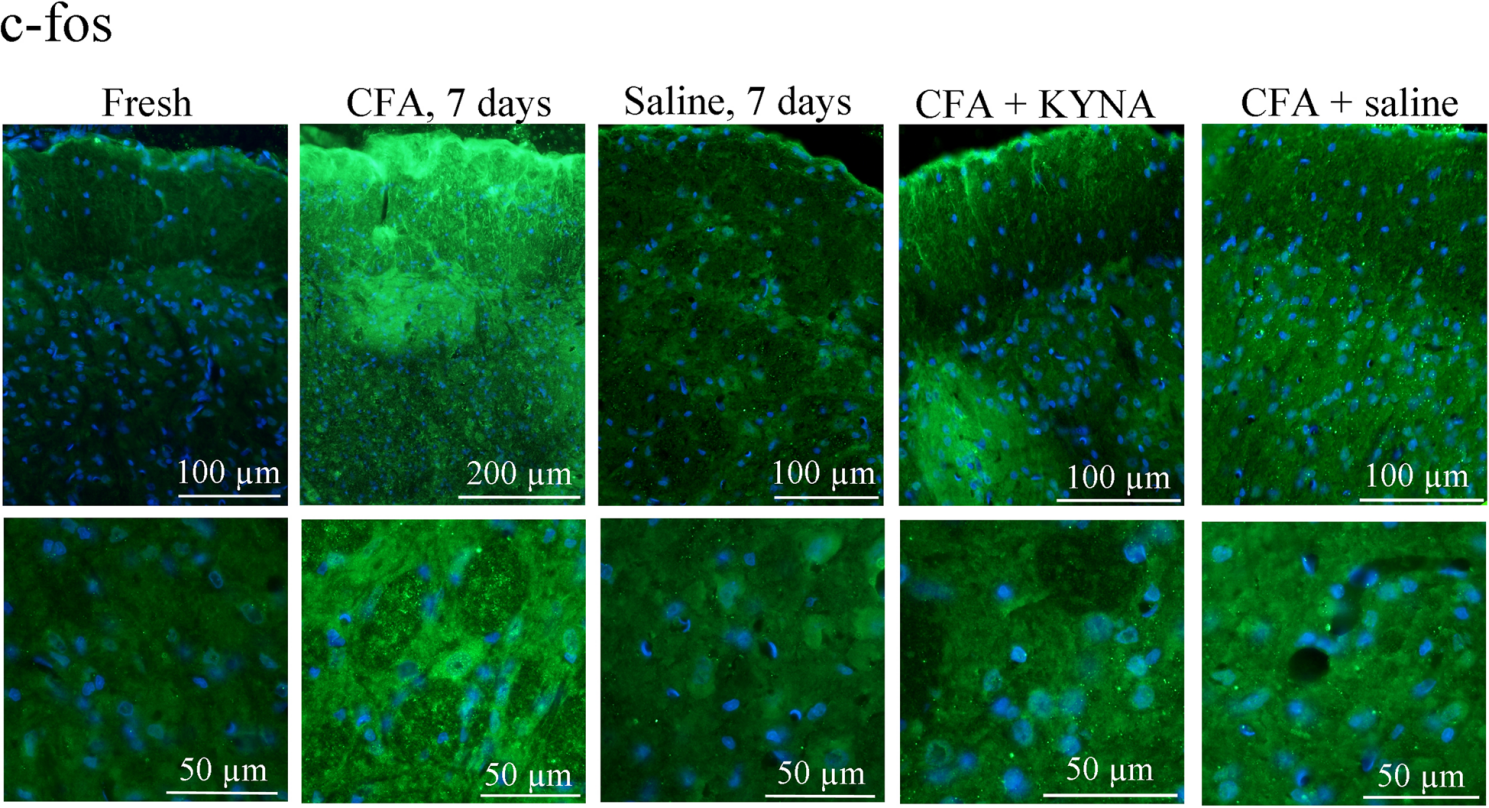
C-fos immunoreactivity in the TNC. In case of fresh, intact rats few c-fos positive neuronal nuclei but no nucleolei could be observed. After appilcation of CFA on t he dura mater large amount of c-fos positive nuclei was detected. Saline application on the dura mater caused no increase in immunoreactivity compared to fresh, intact control. I.p. treatment with KYNA derivate was able to abolish the activation caused by application of CFA on the dura. I.p. saline had no such effect. “+” represents a light, “++” moderate, “+++” strong increase in immunoreactivity

In intact (fresh) rats, few c-fos positive neuronal nuclei, but no nucleolei, were observed in the caudal part of the spinal trigeminal nucleus (Sp5C, TNC); these were mainly seen in the gelatinous layer (Fig. 2). No difference could be observed between cranial and caudal levels of the TNC.

After application of CFA onto the dura mater, an increase in the number of c-fos positve nuclei could be detected, especially in the caudal areas of the TNC, close to the spinal cord (Fig. 2). No increased immunoreactivity was visualised using saline application on the dura (Fig. 2).

Administration of SZR72 reduced the CFA-induced activation in neuronal nuclei at every level of the TNC, similar to the low expression seen in fresh rats. No significant difference could be shown between the pre-treatment and repeated-treatment of SZR72. After treatment with saline, we noted no increase in the c-fos expression, showing that treatment with saline did not have effect on the CFA-induced TNC activation (Fig. 2i).

The results of the c-fos immunostaining are summarized in Table 5.

**Table 5:** Summary of c-fos immunostaining in TNC for different treatment groups

Group	Neuronal staining
Fresh	-\+
CFA 7d	+++
Saline 7d	+
CFA + SZR72 one dose	+
CFA+ SZR72 repeated	+
CFA + saline	+++
CFA + saline repeated	+++

#### PACAP

PACAP immunoreactivity was found in fibers of the trigeminal tract, both in healthy and CFA inflammation-induced animals. PACAP immunoreactivity was found in the brainstem, in the spinal cord, especially in the large neurons of the anterior horn, in the dorsal horn, around the central canal and the ependymal cells of the central canal. PACAP immunoreactive fibers were observed in almost every tract in the spinal cord (dorsal corticocerebellar tract, spinocerebellar tracts, medial longitudinal tract, pyramidal tract). In these territories no difference was detected between different groups (SZR 72 had no effect).

#### Substance P

**Fig. 3:**
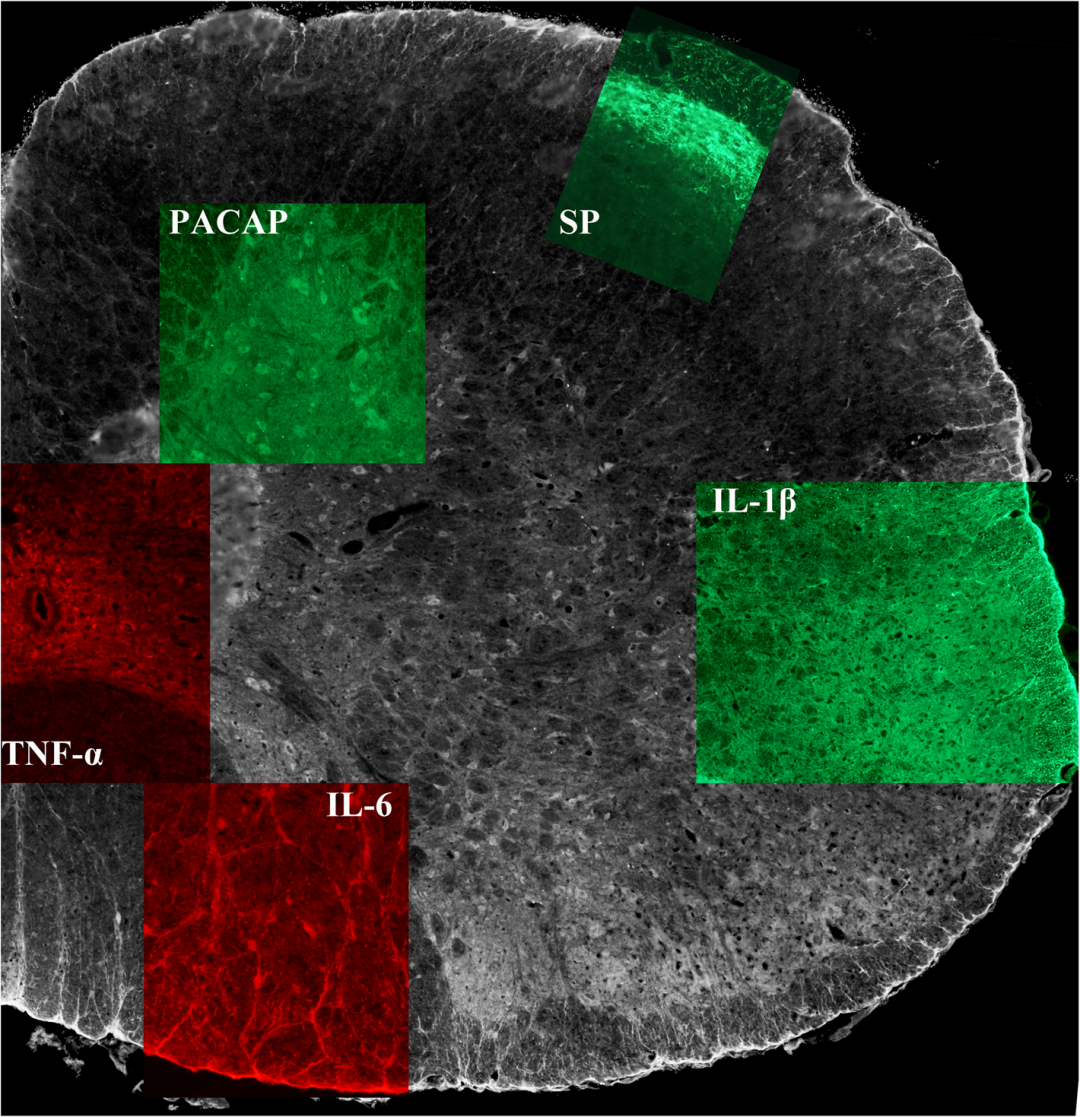
Summary of PACAP, Substance P, TNF-α, IL-6 and IL-1β immunoreactivity in the C_1_-C_2_ region of the spinal cord. PACAP immunoreactivity was detected in the dorsal horn and in almost every tract of the spinal cord. Substance P immunoreactvity was limited to the fibers of the spinal trigeminal tract. TNF-α immunopositivity was mainy seen in the small cells surrounding the central canal. IL-6 immunopositivity was shown in the large neurons of the anterior horn and in nerve endings of these cells. IL-1 β immunopositvity was mainy seen in different tracts of the spinal cord, few positive cells, with granular intracytoplasmatic staining were also detected

Substance P immunoreactivity was limited to nerve fibers of the spinal trigeminal tract and to the gelatinous layer (Fig. 3). Some positive fibers, surrounding the SP5C were also visualized. No difference was noted between different levels of TNC and a slight increased intensity of the fiber staining could be detected after CFA application, but not in the gelatinous layer.

#### TNF-α

TNF-immunoreactivity was found in the trigeminal tract, with dense fiber staining in the spinal trigeminal tract, but no glial or neuronal staining was detected at either level of the TNC. In the spinal cord few, small sized neurons were detected, especially around the central canal (Fig. 3). Some TNF- α positive fibers were identified in other tracts of the spinal cord (dorsal cortico-cerebellar tract, spino-cerebellar tracts, medial longitudinal tract, pyramidal tract). No difference was noted between the groups.

#### IL-6

IL-6 positivity was detected in the fibers and in the cytoplasm of some glial cells, showing a homogenous staining in the spinal trigeminal tract. Some homogeniously stained neurons were detected in the TNC. In the spinal cord, some positive neurons could be seen in the caudal part of the spinal trigeminal nucleus, in the large neurons of the anterior horn, in the dorsal horn and the ependymal cells of the central canal (Fig. 3). Intensely positive fibers were visualised in the cuneate and gracile fasciculus. After application of CFA, similar staining patterns as for the non-CFA groups were found.

#### IL-1β

IL-1β immunohistochemistry showed the same staining pattern as for IL6 and TNFα, with no change after CFA induced activation (Fig. 3). IL-1β immunoreactivity showed a granular cytoplasmatic stainig, previously described in the TG (14).

## Discussion

In this study we present the immunostaining pattern of several neuronal messengers and cytokines in the TNC/C_1_-C_2_ spinal region (11) that are indicated in migraine pathophysiology. CFA is a potent immun- potentiator, used in various peripheral pain model. (Spinal distribution of c-Fos activated neurons expressing enkephalin in acute and chronic pain models, 1^st^ manu) We asked the question whether application of CFA on a defined area of the dura mater could cause activation of second-order neurons in the TNC and whether this activation can be mitigated by systemic adminstration of a KYNA analogue.

Gluatamate, the major excitatory neurotransmitter in CNS plays a key role in the trigeminovascular activation, especially in central sensitisation via activation of NMDA receptors [18, 19]. Glutamate appears to be involved in nociception since glutamate is expressed in the trigeminal ganglion and other sensory ganglia [20, 21]. Glutamate can be released from neurons following nociceptive stimuli putatively acting on satellite glial cells (SGC) [22] but is also expressed in the sensory Aδ – fibers (19).

The kynurenine pathway, the major route of tryptophan metabolism to nicotinamide, has an important role in several diseases of the CNS [23–25]. Kynurenic acid (KYNA) is one of the neuroactive metabolits of the kynurenine pathway in human astrocytes [26] protecting against neuronal cell-death [27]. KYNA in low concentration enhances AMPA receptor activity [28, 29], while in high concentrations blocks the NR1 subunit of the NMDA receptors [19, 30]. NMDA receptor consists of NR1, NR2 and NR3 subunits, where NR1 subunit has a glycine- binding domain. Glycine is essential for the functioning of the NMDA receptor and KYNA acts as an antagonist on the gylcine-binding site (NR1) (18). High level of KYNA might have a neuroprotective effect and could act on glutamate receptors, exerting an inhibitory effect on glutamate release [23].

Here we asked the question whether the KYNA analogue might act on the fibers and cells in the TNC/C_1_-C_2_. Previous work has shown a positive effect in TG following CFA injection into the temporomandibular joint [31] while SZR 72 decreased c-fos activation in the TNC in nitroglycerin induced trigeminal activation [32]. We have reported that one dose of SZR 72 is able to reduce dura mater applied CFA induced activation in the TG [16]. C-fos immunoreactivity is a widely used marker of neuronal activity in the TNC [33, 34]. In the present study we report increased c-fos immunoreactivity following dura mater application of CFA as a sign of neuronal activity of TNC neurons. This effect is attenuated by SZR72. Glutamate activation as a sign of central sensitization can be observed in the second-order neurons after use of CFA, this effect that is also mitigated by the KYNA analogue. Surprisingly repeated-treatment of SZR 72 was not seen to be more effective than pre-treatment with one dose prior CFA application neither in the TG [16] nor in the TNC. Therefore we postulate that early KYNA derivate intervention can block the development of central sensitization, whereas late, repeated treatment might not be able to further moderate mechanisms of central sensitization. Consequently, we assume that the action of the KYNA analogue seems to be exerted on the periphery that is conveyed to neurons of TNC, but an effect on central mechanisms cannot be surely excluded. Further studies are needed to elucidate the possible site of actions of the KYNA derivate.

In this study we examine a fair number of molecules suggested to play a role in migraine. Among these CGRP and PACAP 38 (PACAP) is currently of particular interest. CGRP plays an important role in migraine pathophysiology and localization of CGRP and its receptors (CLR and RAMP1) has already been described in TNC and C_1_ region of the spinal cord [35, 36]. PACAP is a neuromodulator that has some common actions with CGRP, sharing the same receptors RAMP1 subunit [37]. PACAP might play a role in migraine having various neurobiological functions such as inhibitory effect on neurogenic inflammation [38]. PACAP has shown to be involved in trigeminovascular activation as PACAP-38 infusion caused headache in healthy volunteers [39] and PACAP-38-like immunoreactivity has proved to be altered in ictal compared to interictal phase of migraine and in cluster headache [40, 41]. It is suggested that the effect of PACAP is biphasic: lower concentration increasing, higher concentration inhibiting the NMDA receptor activation [42]. We have found PACAP positive but no change in TNC and C_1_-C_2._

In addition, we examined several other molecules putatively involved in migraine pathophysiology: SP, IL-1β, IL-6 and TNF-α which have all been shown to be associated with activation of the trigeminovascular system [43–46]. While we could document their presesnce in the TNC and C_1_-C_2_ of the spinal cord, we did not observe a difference in expression between saline vehicle or CFA administration.

## Conclusion

In conclusion, we report activation in neurons and fibers of TNC and C_1_-C_2_ following application of CFA on the dura mater that was mitigated by SZR 72. To our knowledge this is the first study that presents the cellular distribution of proinflammatory cytokines (IL-6, IL- 1β and TNF-α) in the TNC and in the C_1_-C_2_ region of the spinal cord. This might represent a further step in understanding the functional neuroanatomy of the trigeminal pathway. We found increased c-fos and glutamate immunoreactivity in the TNC following application of CFA on the dura mater that was abolished by the KYNA derivate. Further studies are needed to explore the possible mechanisms involved, which could result in a new therapeutic line in treatment of migraine.
